# The effect of thermal dose on hyperthermia-mediated inhibition of DNA repair through homologous recombination

**DOI:** 10.18632/oncotarget.17861

**Published:** 2017-05-15

**Authors:** Nathalie van den Tempel, Charlie Laffeber, Hanny Odijk, Wiggert A. van Cappellen, Gerard C. van Rhoon, Martine Franckena, Roland Kanaar

**Affiliations:** ^1^ Department of Molecular Genetics, Cancer Genomics Center Netherlands, Erasmus University Medical Center, 3000 CA, Rotterdam, The Netherlands; ^2^ Optical Imaging Center, Department of Pathology, Erasmus University Medical Center, 3000 CA, Rotterdam, The Netherlands; ^3^ Department of Radiation Oncology, Erasmus MC Cancer Institute, 3008 AE, Rotterdam, The Netherlands

**Keywords:** hyperthermia, thermal dose, homologous recombination, BRCA2, RAD51

## Abstract

Hyperthermia has a number of biological effects that sensitize tumors to radiotherapy in the range between 40-44 °C. One of these effects is heat-induced degradation of BRCA2 that in turn causes reduced RAD51 focus formation, which results in an attenuation of DNA repair through homologous recombination. Prompted by this molecular insight into how hyperthermia attenuates homologous recombination, we now quantitatively explore time and temperature dynamics of hyperthermia on BRCA2 levels and RAD51 focus formation in cell culture models, and link this to their clonogenic survival capacity after irradiation (0-6 Gy). For treatment temperatures above 41 °C, we found a decrease in cell survival, an increase in sensitization towards irradiation, a decrease of BRCA2 protein levels, and altered RAD51 focus formation. When the temperatures exceeded 43 °C, we found that hyperthermia alone killed more cells directly, and that processes other than homologous recombination were affected by the heat. This study demonstrates that optimal inhibition of HR is achieved by subjecting cells to hyperthermia at 41-43 °C for 30 to 60 minutes. Our data provides a guideline for the clinical application of novel combination treatments that could exploit hyperthermia's attenuation of homologous recombination, such as the combination of hyperthermia with PARP-inhibitors for non-*BRCA* mutations carriers.

## INTRODUCTION

Hyperthermia is an anti-cancer treatment during which external heat sources are employed to treat tumors. During the treatment, specialized equipment is used to regionally heat the tumor to a final temperature in the range of 40-44 °C [1], which is a safe and effective way to enhance the effectiveness of radiotherapy and some types of chemotherapy, such as cisplatin, carboplatin, cyclophosphamide, ifosfamide, melphalan and mitomycin C [2, 3]. Hyperthermia's sensitization effects towards radiotherapy and chemotherapy can be attributed to a plethora of biological effects in the tumor, both on a macroscopic and microscopic scale. Research that aims to elucidate the biological effects of heat has the potential to revolutionize the way in which hyperthermia will be employed in a clinical setting [4–8].

One of the effects of hyperthermia described more recently is the induction of degradation of the BRCA2-protein [9]. BRCA2 is essential for repair of DNA double strand breaks via homologous recombination (HR) [10]. HR faithfully restores these breaks by copying the information from an intact copy of the damaged DNA, a process catalyzed by the protein RAD51 [11]. BRCA2 is necessary for the loading of RAD51 onto DNA breaks and by degrading BRCA2, hyperthermia causes aberrant localization of RAD51 [9]. This, in turn, causes the attenuation of DNA repair via HR, which at least partly explains hyperthermia's sensitizing effects towards radiotherapy, the latter being based on the creation of an overload of cytotoxic DNA breaks in tumor cells. DNA repair pathways can counteract the cytotoxicity, and thus, by degrading BRCA2, hyperthermia provides a window of opportunity to leave DNA damage unrepaired. Moreover, hyperthermia-mediated BRCA2 degradation creates specific opportunities to increase treatment efficacy, because it induces a localized environment of HR-deficiency. This could potentially be exploited by new treatment regimens that reduce cancer treatment side-effects, such as combination of hyperthermia with new classes of indirect double-strand break inducing agents, like PARP-inhibitors [12, 13].

Before exploiting hyperthermia-mediated atten-uation of HR in a clinical setting, it is important to understand the dynamics of BRCA2 degradation and HR-efficiency upon exposures to different temperatures and treatment lengths, or, thermal doses. By using a set of *in vitro* experiments, we systematically investigated the effects of various thermal doses (ranging from 40-44 °C for up to four hours) on HR-parameters such as BRCA2 degradation and RAD51 focus formation, and we examined the extent of cellular sensitization towards radiation. Our findings provide insight into threshold and saturation levels of BRCA2 degradation upon heat treatment and thereby give insight into the relation between thermal dose and HR-efficiency.

## RESULTS

### Thermal dose is a determinant for radiosensitisation

To investigate the influence of thermal dose on HR, we first established the thermal doses to be used in this study (Table 1). Because we are interested in the influence of thermal doses currently achieved and aimed for in a clinical setting, we selected them based on temperatures in the range of 40-44 °C, and on the current duration of 60 minutes. Additional lengths of treatment were chosen to determine an optimum dose for HR-inhibition.

**Table 1:** Contains an overview of the thermal doses employed in this study

Temperature	Time 0 (37 °C)	Time 1	Time 2	Time 3
**40 °C**	0 min	60 min	120 min	240 min
**41 °C**	0 min	60 min	120 min	240 min
**42 °C**	0 min	30 min	60 min	120 min
**43 °C**	0 min	15 min	30 min	60 min
**43.5 °C**	0 min	15 min	30 min	60 min
**44 °C**	0 min	15 min	30 min	60 min

**Figure 1:**
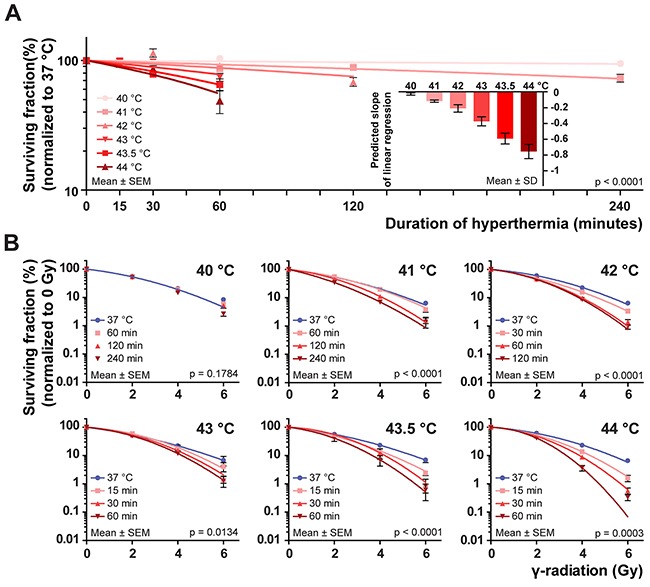
Plating efficiency and colony cell survival at different thermal doses Four different cell lines (BLM, HeLa, FaDu and VH10-SV40) were submitted to hyperthermia and irradiated afterwards in six independent experiments; one for each temperature. The data points and error bars represent mean ± SEM of all pooled cell lines and the connecting curve is the result of a fitted regression. Multiple curves were predicted if the regression parameters differed significantly from each other (p<0.05). Exact p-value is embedded in the graphs. **(A)** Plating efficiencies at 0 Gy for all temperatures, normalized to 37 °C. Curves were predicted by linear regression and the inset graph shows the predicted slope of the regression analysis (Mean ± SD). **(B)** Surviving fractions upon irradiation, normalized to 0 Gy for each thermal dose. The curves are predictions made by linear-quadratic regression. At 40 °C, the single curve explains the variation in the data. Between the experiments, one regression line was predicted to explain survival towards irradiation at 37 °C. Clonogenic survival curves for the individual cell lines can be found in Supplementary Figure 1.

To provide a framework for the assays in which we will determine inhibition of HR by hyperthermia, we established the ability of these selected thermal doses to kill cells directly, and determined their capability to sensitize cells to irradiation. To get a general overview of the biological principles that guide inhibition of HR by hyperthermia, we combined the results of four different cell lines. We started by establishing colony survival curves of three different cell lines that represent various cancer types that are treated with hyperthermia: BLM (melanoma), HeLa (cervix) and FaDu (head and neck), and a cell line that represents a p53-negative tumor: simian virus 40-immortalized fibroblasts (VH10-SV40). We treated the cells with the selected thermal doses and with doses of radiation ranging from 0 – 6 Gy. Consistent with previous studies [14–16], the colony plating efficiency for each cell line was reduced after treatment with temperatures higher than 40 °C (Figure 1A), demonstrating hyperthermia's ability to kill cells directly.

To closely examine radiosensitisation by hyper-thermia, we normalized each colony survival curve belonging to a certain thermal dose for plating efficiency at 0 Gy to 100% (Supplementary Figure 1). We then combined the data points from all four cell lines, and fitted a linear-quadratic curve to the pooled data (Figure 1B) [17]. This analysis resulted in an overview of the response to heat and irradiation in the employed cell lines: hyperthermia at 40 °C failed to increase sensitivity to irradiation, indicated by the prediction of one curve to explain the data variation [17]. For all thermal doses employing temperatures higher than 41 °C multiple curves were obtained, indicating these thermal doses sensitized the tumor cells to irradiation. The maximum extent of radiosensitisation at 41 °C was reached after two hours of treatment, while the same effect was already reached after one hour at 42 °C. Doubling the treatment times did not increase radiosensitisation any further, indicating a saturation of the effects of heat over time. The time-dependent saturation was not observed when cells were treated with 43 °C, 43.5 °C or 44 °C (Figure 1B).

### Temperatures higher than 40 °C cause significant degradation of functional BRCA2 proteins

**Figure 2:**
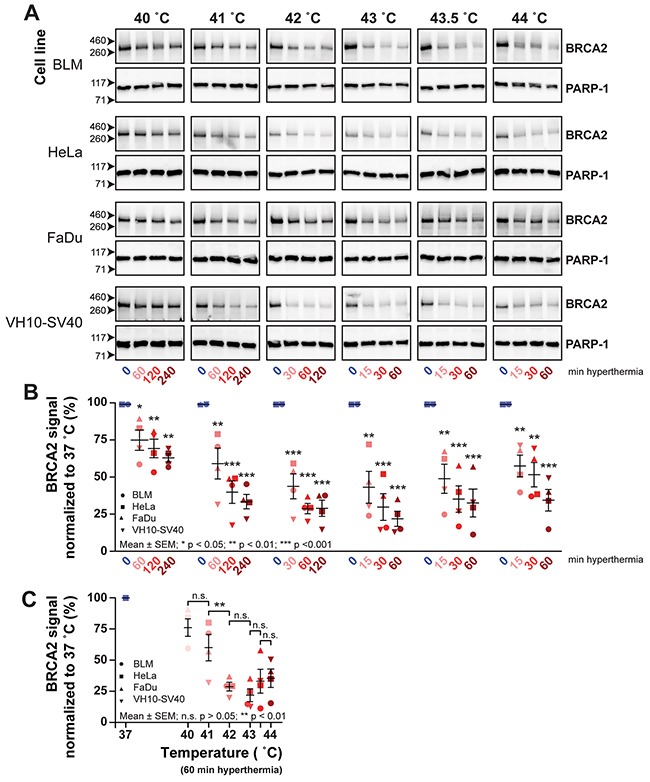
The BRCA2 protein is degraded at temperatures higher than 40 °C Cells from four different lines BLM (circle), HeLa (square), FaDu (triangle), VH10-SV40 (inverted triangle) were submitted to hyperthermia and subsequently lysed in six separate experiments; one for each temperature. **(A)** BRCA2-signals of all separate samples on cropped immunoblots. PARP-1 is used as a loading control. **(B)** Quantification of BRCA2-protein signals from **A**. Each hyperthermia-treated sample is represented as a percentage of the BRCA2 signal at 37 °C. The error bars denote mean ± SEM. The statistical differences relative to 37 °C were determined by ANOVA and followed by Tukey's Multiple Comparison Test. **(C)** Quantification of BRCA2 after 60 minutes of a given temperature. Statistical differences were determined by ANOVA and followed by Tukey's Multiple Comparison test.

Hyperthermia at 42.5 °C inhibits HR by inducing proteasomal degradation of the BRCA2 protein [9]. To study the effects of thermal dose on HR, we started by examining the ability of the selected thermal doses to induce degradation of the BRCA2 protein, by measuring the BRCA2-protein levels in whole cell extracts from the four cell lines treated with the thermal doses by immunoblot (Figure 2A). We quantified the BRCA2-signals on the immunoblot and normalized these to the signal at 37 °C (Figure 2B). Although each thermal dose had an effect on BRCA2 protein level, the extent of the effect was quite different. For the four cell lines tested, the lowest mean BRCA2 protein level (22%) was reached after 60 minutes treatment at 43 °C, while as much as 64% of the BRCA2 signal remained after four hours of treatment at 40 °C (Figure 2B). This indicates that BRCA2 degradation is dependent on the applied thermal dose, and presumably, that treatment at 40 °C might be insufficient to achieve a significant reduction in BRCA2 protein levels and thereby in HR. Upon examination of the BRCA2 protein levels after treatment for 60 minutes at the different temperatures, we noticed the most significant decrease in the level between 41 °C and 42 °C (Figure 2C). Interestingly, when temperatures surpassed 42 °C, the degradation of BRCA2 observed in the whole cell lysates seemed similar or even less efficient than at 42 °C itself, which is especially pronounced in FaDu cells (Figure 2C).

**Figure 3:**
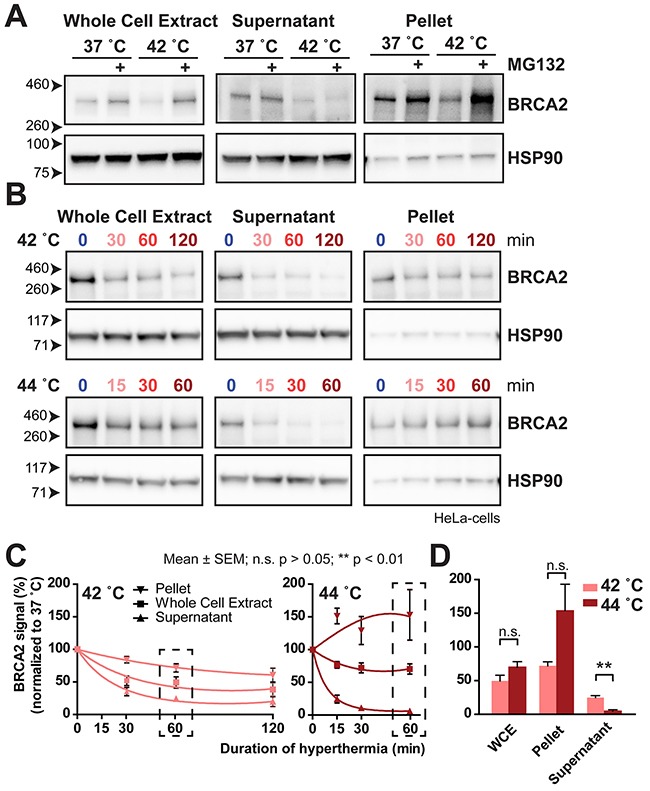
BRCA2 moves to an insoluble fraction after treatment with MG132 or 44 °C **(A)** BRCA2 protein levels at 37 °C and 42 °C in HeLa cells treated with or without 50 μM MG132 on cropped immunoblots. Signals shown are from the whole cell extract (left), and from two fractions after centrifugation: the supernatant (middle) and in the pellet fraction (right). HSP90 is used as a loading control. **(B)** BRCA2 protein levels on cropped immunoblots at 42 °C (upper panel) and 44 °C (lower panel) in HeLa cells in the whole cell extract (left), and from the two fractions after centrifugation: the supernatant (middle) and in the pellet fraction (right). HSP90 is shown as a loading control. **(C)** Quantification of BRCA2 signals in **B**, corrected for the 37 °C control. A linear-quadratic regression indicates three separate curves for each series of measurements, indicating a significant difference in BRCA2-protein levels in the whole cell extract, supernatant and pellet. The error bars denote mean ± SEM obtained in three experiments. **(D)** Bars zoom in on the 60-minute treatment point in **C**. Statistical differences of column pairs were determined using a student's *t*-test.

We therefore investigated whether the BRCA2-signal in the whole cell extract at temperatures exceeding 43 °C represents a functional pool of the BRCA2 protein. Since degradation of BRCA2 is mediated by the proteasome, a heat-mediated malfunction of proteasomes could lead to a failure to detect declining BRCA2-signals in a whole cell extract. When the proteasome inhibitor MG132 is added to cells previous to hyperthermia treatment, the BRCA2-protein levels are rescued in a whole cell extract [9]. However, when the cell lysates from cells treated with MG132 and hyperthermia are fractionated, the entire fraction of protected BRCA2 is found in the pellet instead of in the supernatant, indicating that the heat caused the protein to become insoluble (Figure 3A). To investigate what happens with the BRCA2-proteins found at 44 °C, we performed the same simple fractionation of HeLa cells treated with the thermal doses employing temperatures of 42 °C and 44 °C (Figure 3B and 3C).

The reduction of the BRCA2 signal in the whole cell extract in this experiment was clearly visible when the cells were treated with 42 °C and 44 °C (Figure 3B). However, cells treated for 60 minutes at 42 °C seemed to have lower BRCA2-levels than cells treated for 60 minutes at 44 °C, confirming our results from Figure 2C. Strikingly, the localization of BRCA2 in the extracts was very different: we found that the BRCA2 levels in the supernatant were lower at 44 °C than at 42 °C, indicating that the higher temperature is a stronger inducer of BRCA2-degradation (Figure 3C and 3D). In contrast to the supernatant, the BRCA2 protein detected in the pellet increased over time when temperature was set from 37 to 44 °C, while it decreased over time when cells were treated with 42 °C (Figure 3C). This finding indicates that although the BRCA2 protein is still present in the whole cell extract at the higher temperatures, it represents a pool of protein that has aggregated in a fraction that ends up in the pellet, and is therefore unlikely to be functional.

### Localization of RAD51 in cells is differentially affected by thermal dose

Because we found different levels of BRCA2 protein in response to different thermal doses, and found that temperatures exceeding 43 °C did not completely eradicate BRCA2 protein levels in the whole cell extract (Figure 3), we investigated the presence of RAD51 foci upon treatment with the different thermal doses. These foci represent the localization of the RAD51-protein onto DNA double strand breaks and are a read-out for effectivity of HR, in particular for the functionality of the BRCA2-protein [18]. Correlating to BRCA2-degradation upon hyperthermia treatment (41-42 °C), RAD51 fails to localize onto double strand breaks in cells treated at these temperatures [9]. To investigate the appearance and behavior of RAD51 foci upon treatment with the various thermal doses, we treated HeLa cells with these doses, irradiated them with 4 Gy, and fixed them either 30, 60 or 120 minutes after the irradiation. HR requires a copy of the damaged DNA, usually the sister chromatid, and is therefore limited to the S- and G2-phase of the cell cycle [11]. Therefore, to confirm the cells’ ability to form RAD51 foci, we used a positive signal for incorporated EdU as a prerequisite for cells to be analyzed.

**Figure 4:**
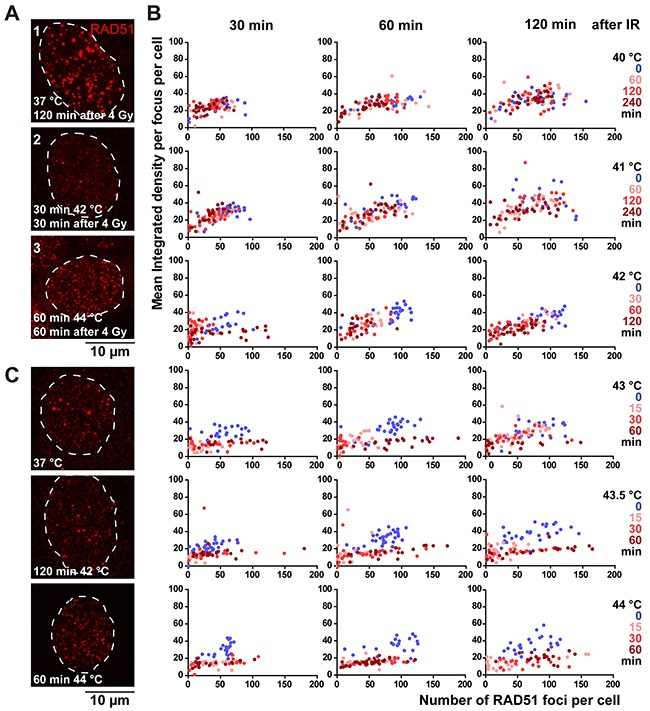
RAD51 foci behave differently depending on the thermal dose **(A)** Representative pictures of RAD51-staining pattern upon 4 Gy irradiation in EdU-positive nuclei of HeLa cells. The RAD51 pattern shown in picture 1 is characterized as ‘normal’. In picture 2 only few foci can be seen, and in picture 3, many, but mostly very small foci can be distinguished. **(B)** 2D-representation of measured focus-structures in EdU-positive cells. Each dot represents a cell, with on the X-axis the number of foci and on the Y-axis the mean integrated density per focus. Thermal doses are indicated with the different colors. A shift of the dot-cloud represents a different appearance or, qualification of the RAD51 focus. **(C)** Representative pictures of RAD51-staining pattern in EdU-positive nuclei in unirradiated cells.

Upon examining the RAD51 signal in the EdU-positive cells, we found three very distinguished foci-patterns, which we refer to as Category 1-3 foci. Cells in Category 1 presented with many large foci, or “normal”, based on our extensive experience [19–21]. Cells with the Category 2 focus pattern displayed fewer, but quite large foci. The Category 3 focus pattern was represented by cells with many, but mostly very small foci (Figure 4A). To objectively describe these focus structures, we designed an image analysis tool that measures several aspects of RAD51 foci in a cell nucleus: the number of foci, their average area and their average intensity (Supplementary Figure 2). Since both area and intensity can be used to classify the response of the foci to temperature, we combined them by multiplication, resulting in the mean integrated density per focus per nucleus. We plotted the integrated density and the number of RAD51 foci per cell in a 2D histogram (Figure 4B). While there is little to no difference between cells that received heat treatment at 40 °C or treatment at 37 °C, cells treated with hyperthermia at the higher temperatures (indicated by the cloud of red points), shifted away from the cells treated at 37 °C (cloud of blue points) (Figure 4B). Compared to cells that were treated at 37 °C, cells that were treated with 41 °C, 42 °C, or, up to 15 minutes with 43 °C or 43.5 °C had mostly fewer foci (Category 2) (Figure 4B). However, cells treated with 44 °C or with more than 15 minutes at 43 or 43.5 °C had foci that were smaller in size and intensity (Category 3) (Figure 4B).

Upon examination of the Category 3-type foci, we noticed that they also were present in cells that had not been irradiated (Figure 4C), indicating that they form spontaneously upon heat treatment. The Category 3 RAD51 foci have been described earlier as a representation of stalled replication forks, which are able to form independently of functional BRCA2 protein [22]. These structures might therefore be additional proof for the hypothesis that hyperthermia with temperatures exceeding 43 °C causes problems in the S-phase of the cells [16]. Concordantly, we found that cells with Category 3 foci had a less intense EdU-signal compared to cells that had normal foci (Category 1) or less foci (Category 2) (Supplementary Figure 2E). The EdU-signal did recover two hours after stopping hyperthermia treatment, hinting that the problems in S-phase could be reversible over time. This combined evidence suggests that the disappearance of the Category 1 RAD51 foci after treatment with temperatures higher than 40 °C indicates inhibited HR, and that the Category 3 foci represent another effect of hyperthermia.

## DISCUSSION

The elucidation of the mechanisms of the biological processes responsible for the sensitizing effect of heat towards radiotherapy and various chemotherapies is an active field of research. Among these processes DNA repair pathways are attractive targets of hyperthermia because their activity modulates the cytotoxicity of DNA breaks in tumor cells, on which the efficacy of radiation and chemotherapy is based [23]. Although many DNA repair pathways are thought to be affected by heat, one pathway is of specific interest in this context: HR. This is because the discovery that hyperthermia can be used to locally and on demand inhibit the activity of the HR DNA repair pathway opens up avenues to novel combination therapies; specifically the combination of hyperthermia with PARP-inhibitors. PARP-inhibitors selective kill HR-deficient cells and are currently clinically applied for patient with HR-defective tumors due to genetic BRCA deficiency [24–26]. Inhibition of HR by hyperthermia carries the promise that PARP-inhibitors can be successfully used in much broader patient populations, as it will temporarily inactive HR, irrespective of the patient's genetic make-up [9, 12].

In this study, we systematically explored the influence of various thermal doses on HR by a set of *in vitro* experiments. We used two parameters of HR-effectivity: BRCA2 protein levels and RAD51 focus formation upon irradiation, and established survival curves at the same thermal doses to relate HR parameters to a functional outcome (Supplementary Table 1). Based on the obtained data employing the thermal doses, we can describe three distinct responses of HR towards hyperthermia.

The first response is a presumed failure to effectively attenuate HR. Cells heated at 40 °C have more than 60% of the BRCA2 protein relative to the non-heated cells, and the formation of RAD51 foci is barely affected by this temperature. This correlates with the finding that hyperthermia at 40 °C does not significantly increase the sensitivity of the cells to irradiation. With respect to the survival results, it should be noted that the average treatment temperatures currently reached in the clinic, ranging between 40 and 41 °C, does strongly enhance treatment outcome [27–32]. This could be explained by two reasons, the first being that the cells in culture are treated with hyperthermia and radiotherapy only once, thus even small, non-significant differences in our colony survival assay could result in larger, significant effects when treatment is repeated multiple times, as is the case in the clinic. The second explanation is that hyperthermia has multiple biological effects, including increased blood flow [33], increased oxidation [34], and activation of the immune system [35], which are obviously not taken into account in our *in vitro* experimental set-up, but might very well mediate the treatment outcome in patients treated with hyperthermia at lower temperatures [5–8].

The second response group is characterized by an attenuation of HR, and presents itself when cells are treated with 41 °C, 42 °C or with 15 or 30 minutes at 43 °C. At 41 °C, 60% of the baseline BRCA2 levels remain after 60 minutes of treatment, and continue to drop with longer treatment times, as do the number and integrated density of RAD51 foci. Moreover, cells are sensitized to irradiation when they are treated for 60 minutes at 41 °C, and this effect can be exaggerated when cells are treated for two hours. However, an additional two hours results only in a small decrease in survival, indicating a saturation of the hyperthermia-mediated effects on the cells. The effects of heat on cell survival and on HR in cells treated with hyperthermia at 42 °C, or for 15-30 minutes at 43 °C are similar to those in cells treated with 41 °C, but more rapid.

The last group within the set of thermal doses employed here encompasses the reaction of cells that are subjected to 60 minutes at 43 °C, or temperatures higher than 43 °C. These thermal doses seem to not only induce HR-deficiency, but affect the cells in many more ways [36]. For example, consistent with previous studies, we find that heat's ability to directly kill cells and sensitize to irradiation is increased, and keeps doing so with longer treatment length [14, 15]. In contrast, BRCA2 protein levels in the whole cell extract cease to decrease over time after the initial drop. However, similar to when cells are treated with a proteasome inhibitor, treatment with hyperthermia at 44 °C causes BRCA2 to accumulate in a pellet fraction, while the amount of BRCA2 in the supernatant cell fraction keeps decreasing over time. This could be explained by possible defects in the functionality of the proteasome, which interferes with the molecular removal of unfolding BRCA2 proteins [37]. Consistent with this reaction, RAD51 focus morphology, the read-out used to determine functionality of BRCA2, alters greatly in this last group. We show that foci become much smaller in size and somewhat less in intensity and that they appear independently of irradiation, indicating they form spontaneously upon heat. These structures resemble RAD51 foci described before to form independent of the BRCA2 protein, and could be stalled replication forks [22]. The possibility that hyperthermia > 43 °C directly affects progression in S-phase has previously been described, and is supported by the low intensity of the EdU-signal in cells treated in our assays, which is indicative of a lack in DNA synthesis [16]. All these findings prove that many more biological mechanisms than HR are affected by temperatures surpassing 43 °C and that these mechanisms result in a lower specificity of the heat treatment.

Concluding, our study demonstrates that if hyperthermia treatment is aimed at optimally inhibiting HR, the temperature which should be strived for is 42 °C. The minimal thermal dose to achieve defects in this DNA repair pathway is 41 °C for one hour, but it is not necessary to surpass 30 minutes at 43 °C. Taking into consideration that in current hyperthermia treatments temperatures higher than 43 °C are rarely reached in the patient [31], our findings can be regarded as reassuring of the current clinical guidelines and possibilities, but can be used to guide technological development of next generation hyperthermia systems. Moreover, it demonstrates that BRCA2 degradation and RAD51 focus formation could both be potential biomarkers for efficiency of hyperthermia treatment. However, the acquired data will be of particular interest as a guideline for potential clinical application of anti-cancer strategies that exploit the heat-mediated attenuation of HR, such as PARP-inhibitors [24, 25] or proton therapy [38, 39], will be combined with hyperthermia in a clinical setting [8, 12].

## MATERIALS AND METHODS

### Experimental set-up, hyperthermia and irradiation

We studied the effects of hyperthermia on HR by exposing cells in 60 mm petri dishes to increased temperatures ranging from 40-44 °C in an incubator with a controlled atmosphere (5% CO_2_ and 20% O_2_) set at the appropriate temperature. Indicated treatment times always exclude the 15 minutes required for the medium to reach the set temperature. The control samples were treated at 37 °C. The cells were exposed to γ-irradiation from a caesium-137 source with a dose rate of 0.64 Gy/min within 15 minutes after hyperthermia treatment.

### Cell culture

The following four human cell lines were used: BLM, HeLa, FaDu and VH10-SV40. All cell lines were cultured in a 1:1 mixture of DMEM (4.5 g/L Glucose, with Ultraglutamine 1) and Ham's F-10 (BioWhittaker™), supplemented with 10% fetal calf serum and 1% penicillin/streptomycin (Sigma-Aldrich), and were maintained in an incubator set at 37 °C and with an atmosphere of 5% CO_2_ and 20% O_2_. Frozen aliquots from same passages were used to minimize experimental variation. The cells were mycoplasma-free and distinguished by morphology.

### Clonogenic assays

Cells were allowed to recover from freezing by being cultured for six days: after thawing on day one, the cells were split on day 2 and were allowed to grow exponentially. Finally on day 5, 24 hours prior to seeding the clonogenic assay, 2*10^6^ BLM or Hela cells and 3*10^6^ FaDu or VH10-SV40 were seeded in 10 cm dishes. At the end of the sixth day, cells were trypsinized, counted with a coulter counter and seeded in triplicates at different concentrations in 60 mm dishes; for the control irradiation (0 Gy), 200 BLM or HeLa cells were seeded, or 300 for either FaDu or VH10-SV40. The amount of seeded cells was doubled for each 2 Gy increase in irradiation dose. The cells were allowed to attach overnight (~14 h) and were treated with the different thermal doses and irradiation the next morning. Cell colonies were allowed to form for 10 days (HeLa and BLM) or 20 days (FaDu and VH10-SV40), after which they were fixed and stained in 45% methanol, 45% dH_2_O, 10% Acetic acid and 0.25% Coomassie Brilliant Blue (Sigma-Aldrich). Colonies containing more than 30 cells were counted using a stereomicroscope.

### Cell lysis and protein assay

The day before the experiment, 0.8*10^6^ (BLM and Hela) or 1.2*10^6^ (FaDu and VH10-SV40) cells were seeded in a 60 mm dish. All cell lysates were made within 30 minutes after hyperthermia treatment, and were in the incubator together with the cells used for clonogenic survival or immunofluorescence analysis. After washing with PBS, the cells were scraped and then lysed in Laemmli sample buffer (2% SDS, 10% Glycerol and 60 mM Tris pH 6.8) and heated at 95 °C for 5 minutes. The sample was passed through a syringe several times to reduce viscosity.

For fractionation experiments, cells in 15 cm dishes were treated with MG132 (Calbiochem) one hour before the start of hyperthermia, or without MG132 at the indicated temperatures. Immediately following treatment, the cells were lysed in NETT buffer (50 mM Tris-HCl pH7.5, 100 mM NaCl, 5mM EDTA 0.5% Triton-X-100, 1x protease inhibitors (Complete, Roche®) and 1 mM pefabloc). After 30 minutes, the cells were scraped and centrifuged at 12000 rpm for 15 minutes at 4 °C. After centrifugation, the pellet and supernatant were separated and the pellet was resuspended in PBS. Laemmli buffer was added to both samples and the mixture was boiled at 95 °C for 5 minutes. Before immunoblotting, the protein concentration was estimated using the Lowry protein assay [40], after which protein samples were prepared by adding loading buffer (final concentration: 0.01% bromophenol blue and 0.5% β-mercaptoethanol).

### Immunoblotting

The samples were run on an SDS-PAGE gel or a 3-8% Tris-Act gel (Novex, ThermoFisher Scientific). Protein transfer on a PVDF membrane was achieved by wet blotting at 300 mA for two hours at 4 °C, using transfer buffer (0.4 M Glycine, 5 mM Tris, 20% Methanol). After transfer, the membrane was blocked in 3% dry skimmed milk in PBS with 0.05% Tween-20. The primary antibody was incubated overnight at 4 °C and the secondary antibody was incubated for 1-2 hours at room temperature. After adding ECL substrate (1:1 mixture of A: 0.1M Tris-HCl pH 8.5, 2.5 mM Luminol, 0.4 mM p-Coumaric acid and B: 0.1 M Tris-HCl pH 8.5, 0.02% hydrogen peroxide), blots were imaged in Alliance 4.7 (Uvitec Cambridge). The antibody signals from the immunoblots were quantified using the ‘Analyze Gels’ tool in FIJI (Image J1.50i, [41]). Equal protein loading was always checked by staining the post-transfer gel with Colloidal Coomassie (0.008% Coomassie Brilliant Blue G-250 and 0.35% glacial HCl in distilled water) by heating for ~25s, shaking for 30 minutes, and subsequent destaining using distilled water.

### EdU incorporation and cell fixation

One day prior to the experiment, 0.4*10^6^ Hela cells were seeded in a coverslip-containing 30 mm dish. The cells were fixed after hyperthermia treatment and irradiation. To distinguish S-phase cells, 10 μM EdU (Invitrogen) was added to the cells 45 minutes prior to fixation. After the indicated time after irradiation, the cells were rinsed with PBS and subsequently incubated for 1 minute in a pre-extraction buffer containing Triton-X-100 (0.5% Triton-X-100; 20 mM HEPES-KOH, pH 7.9; 50 mM NaCl; 3 mM MgCl_2_ and 300 mM sucrose) [42]. After rinsing with PBS, the cells were fixed by incubating them for 15 minutes in 4% paraformaldehyde in PBS, followed by another wash in PBS.

### Immunofluorescence

EdU was detected with the use of a Click-IT® reaction. First the cells were permeabilized with 0.5% Triton-X-100 for 30 minutes, then they were washed twice with 3% BSA in PBS. Next, the cells were incubated for 45 minutes at room temperature in a cocktail containing a final dilution of 43 mM Tris-HCl pH 7.5, 1.6 mM CuSO_4_·5H_2_O, 25 μM ATTO 390 Azide (ATTO-TEC GmbH) and 1 mM Ascorbic Acid. Before labelling RAD51, the cells were washed once more with 3% BSA and continued by a wash step (three times a short wash and two times 10 minutes incubation with PBS 0.1% Triton-X-100), followed by 30 minutes blocking in PBS+ (0.5% BSA and 0.15% Glycine in PBS), overnight incubation at 4 °C with the first antibody in PBS+, another wash step, incubation with secondary antibody for two hours at room temperature. After another wash step and a short wash in PBS only, the samples were embedded in Vectashield (Vector Laboratories) and sealed with nail polish.

### Antibodies

For immunoblotting, the following antibodies and dilutions were used: mouse anti-BRCA2 (1:1000, OP95, Ab-1, Merck Millipore), mouse anti-PARP-1 (1:5000, C2-10, Enzo Lifesciences), mouse anti-HSP90 (1:1000, AC88, Abcam) and HRP-conjugated Sheep anti-mouse IgG (H+L) (1:2000, Jackson ImmunoResearch). For immunofluorescence rabbit anti-RAD51 (1:10000, [43]) and an Alexa Fluor® 594 goat anti-mouse (1:1000) were used.

### Image acquisition and foci counting

The images were obtained with a Leica TCS SP5 confocal microscope, using the 63x oil immersion (n.a. 1.4) objective with an image size of 1024 × 1024 pixels and 82 × 82 μm. Per coverslip, at least four areas that contained EdU-positive cells were imaged in Z-stacks with 14 slices and an increment of 1 μm. Before image analysis, maximum projections were made using FIJI image analysis software (Image J1.50i, [41]). For quantification of foci a homemade image analysis macro within FIJI was used. In short, regions of interest (ROIs) in the EdU-channel were selected, and within these ROIs a threshold was set for RAD51 positive spots using the MaxEntropy algorithm. The ‘Analyze Particles’ function was used to count particles with a minimum size of 0.05 nm and a maximum size of 5 nm.

### Statistics

All graphs and statistical analyses were generated in GraphPad Prism 6.0. Statistical tests for each experiment can be found in the figure legends.

## SUPPLEMENTARY MATERIALS FIGURES AND TABLE





## Abbreviations

HR: homologous recombination
ROI: region of Interest
