# Supplementary material for: The effect of thermal dose on hyperthermia-mediated inhibition of DNA repair through homologous recombination
Source: Oncotarget. 2017 May 15;8(27):44593–604. doi: 10.18632/oncotarget.17861 (PMC5546504; doi:10.18632/oncotarget.17861)
Supplement: Supplementary file 1 [file oncotarget-08-44593-s001.pdf]

The effect of thermal dose on hyperthermia-mediated inhibition of DNA repair through homologous recombination

SUPPLEMENTARY MATERIALS

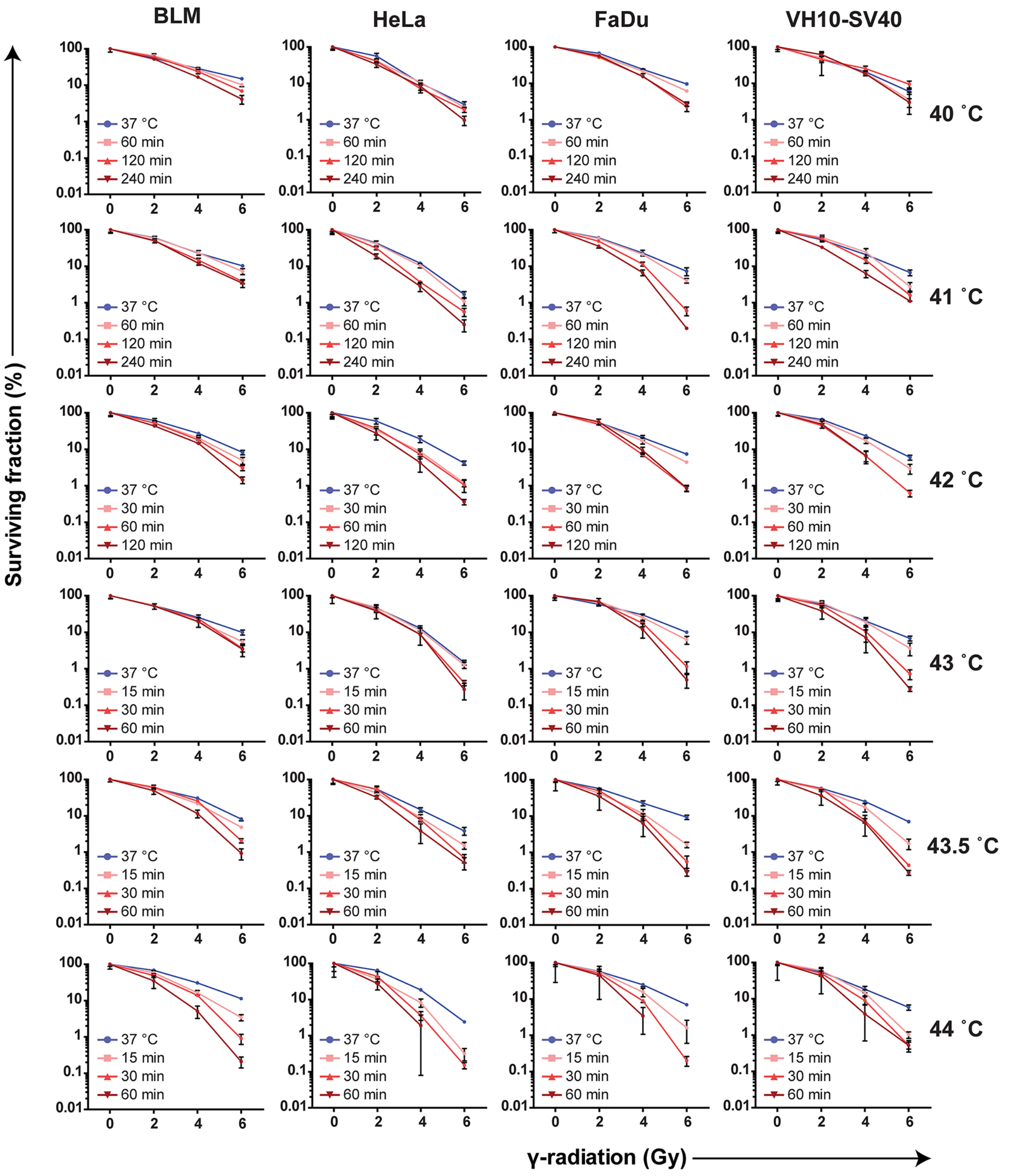

Supplementary Figure 1: Colony cell survival at different thermal doses. Colony survival curves of four cell lines (BLM, HeLa, FaDu and VH10-SV40) submitted to hyperthermia and irradiated afterwards in six independent experiments; one for each temperature. The points and bars represent mean ± SEM of triplicate measurements.

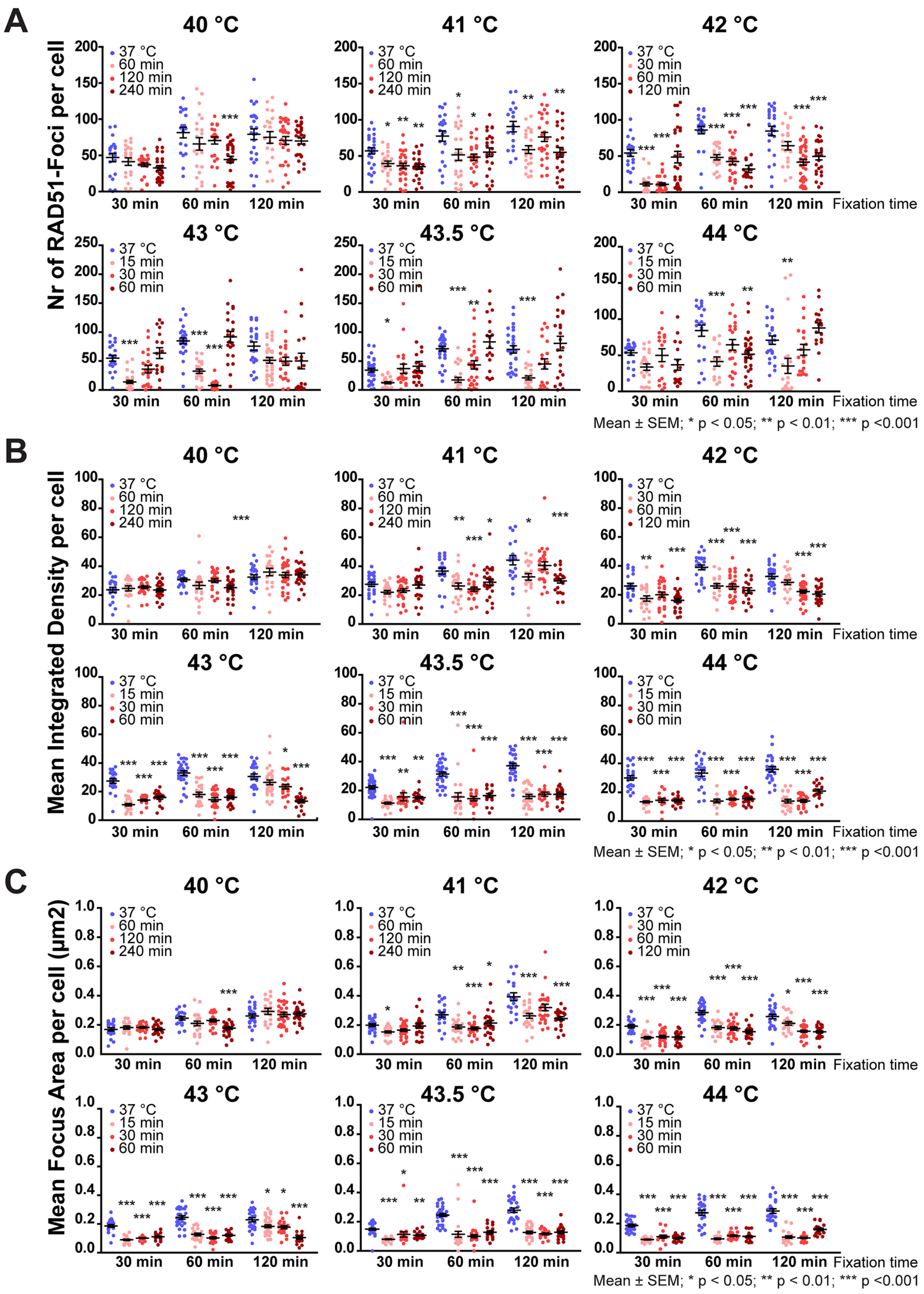

Supplementary Figure 2: Quantification of RAD51 foci upon heat. Each dot in the graphs represents a EdU-positive cell that was submitted to hyperthermia and irradiated with 4 Gy directly afterwards, either fixed 30, 60 or 120 minutes after irradiation (X-axis) and stained for EdU and RAD51. The bars represent mean ± SEM for each thermal dose and fixation time. Several qualities of RAD51 foci and cells were measured: **(A)** number of foci per cell, **(B)** mean integrated density per cell, **(C)** mean focus area per cell. (*Continued*)

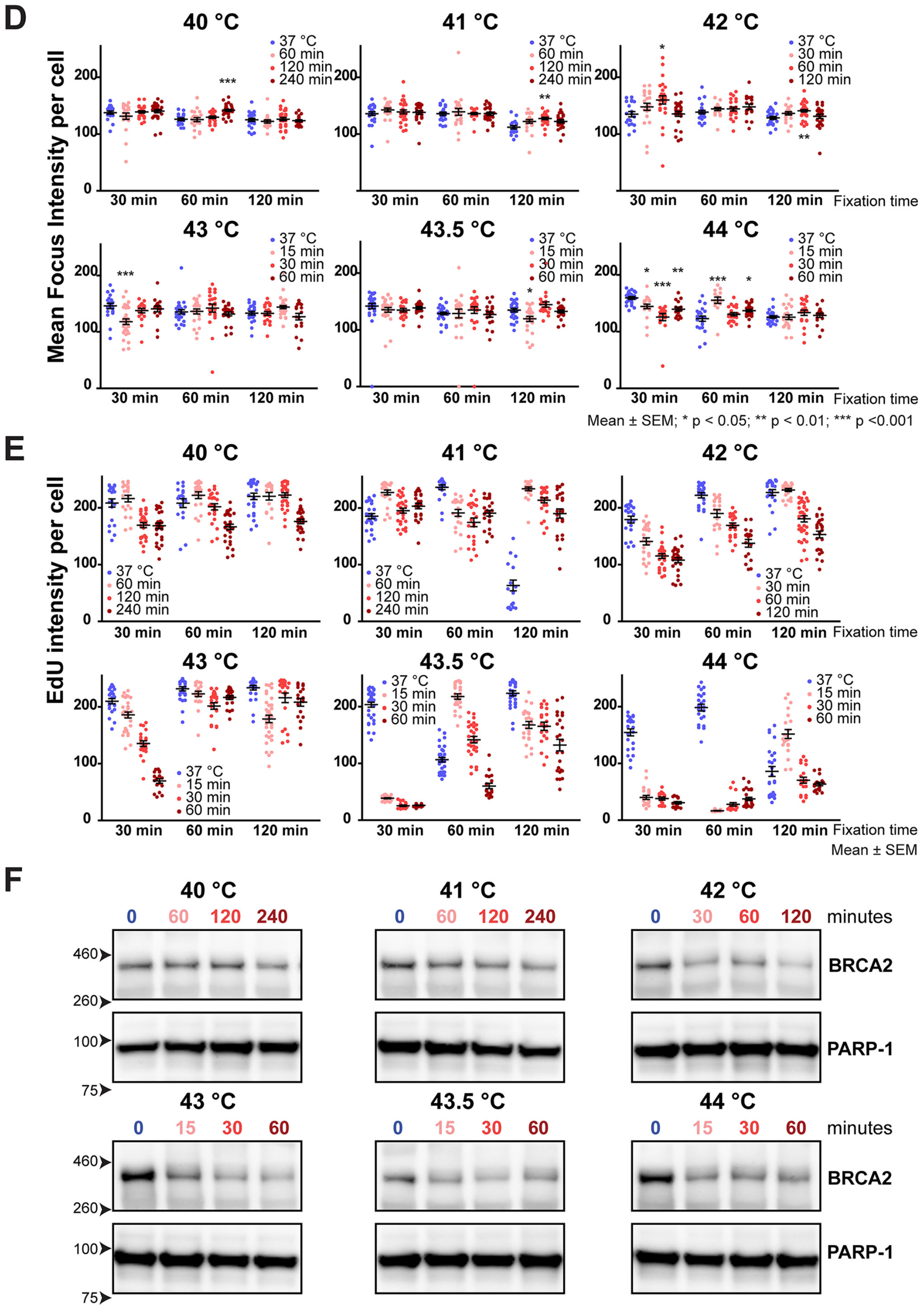

Supplementary Figure 2: (*Continued*) Quantification of RAD51 foci upon heat. (D) Mean focus intensity per cell. The statistical differences, indicated by asterisks, were determined by ANOVA followed by Tukey's Multiple Comparison Test. **(E)** EdU intensity per cell. Statistical differences are not given, some samples had a less intense EdU staining due to experimental variation and artefacts. However, the low EdU intensity at 43, 43.5 and 44 °C can be attributed to effects of hyperthermia, since they are consistent with each other. **(F)** Protein samples made from cells that were incubated at the different thermal doses simultaneously with the cells stained for RAD51. Immunoblot is a cropped representation of probing for BRCA2 and for PARP-1 as a loading control and confirms the effectiveness of BRCA2 degradation by heat.

Supplementary Table 1: Summary of all measured outcomes.

See Supplementary File 1:
