# Supplementary material for: The effect of thermal dose on hyperthermia-mediated inhibition of DNA repair through homologous recombination
Source: Oncotarget. 2017 May 15;8(27):44593–604. doi: 10.18632/oncotarget.17861 (PMC5546504; doi:10.18632/oncotarget.17861)
Supplement: Supplementary file 2 [file oncotarget-08-44593-s002.docx]

| **Temperature** | **Duration of treatment** | **CEM43^a^** | **Cell survival at 0 Gy^b^** | **Relative survival fraction at 4 Gy^c^** | **Relative BRCA2 protein level^d^** | **Relative RAD51 foci number ^e^** | **Relative RAD51 integrated density ^f^** |
| --- | --- | --- | --- | --- | --- | --- | --- |
| 40 °C | 60 min | 8 min | 104% | 40% | 76% | 81% | 87% |
|  | 120 min | 15 min | 93% | 37% | 70% | 86% | 98% |
|  | 240 min | 30 min | 95% | 37% | 64% | 54% | 83% |
| 41 °C | 60 min | 15 min | 87% | 33% | 60% | 66% | 72% |
|  | 120 min | 30 min | 88% | 34% | 41% | 62% | 66% |
|  | 240 min | 60 min | 73% | 28% | 34% | 71% | 79% |
| 42 °C | 30 min | 15 min | 114% | 39% | 44% | 56% | 67% |
|  | 60 min | 30 min | 91% | 32% | 29% | 49% | 66% |
|  | 120 min | 60 min | 68% | 25% | 29% | 37% | 60% |
| 43 °C | 15 min | 15 min | 92% | 39% | 44% | 39% | 55% |
|  | 30 min | 30 min | 97% | 41% | 30% | 9% | 44% |
|  | 60 min | 60 min | 74% | 32% | 22% | 109% | 49% |
| 43.5 °C | 15 min | 21 min | 99% | 38% | 50% | 24% | 50% |
|  | 30 min | 42 min | 78% | 29% | 36% | 61% | 45% |
|  | 60 min | 85 min | 65% | 26% | 33% | 116% | 52% |
| 44 °C | 15 min | 30 min | 94% | 42% | 60% | 49% | 45% |
|  | 30 min | 60 min | 86% | 37% | 53% | 76% | 45% |
|  | 60 min | 120 min | 49% | 22% | 36% | 61% | 46% |

**Supplementary Table 1: Summary of all measured outcomes.**

^a^ CEM43: Cumulative Equivalent Minutes at 43 °C, a dosimetric unit which is commonly used to convert time-temperature doses into a single equivalent dose at 43 °C; in this case calculated based on radiosensitisation parameters (*R* = 2 for temperatures higher than 43 °C; *R* = 0.5 for temperatures lower than 43 °C) [1,2]

^b^ Mean percentage cell survival after hyperthermia alone, normalized to treatment at 37 °C. Full survival curves are presented in Figure 1A.

^c^ Mean percentage cell survival after 4 Gy irradiation, normalized to unirradiated cells. Full survival curves are presented in Figure 1B.

^d^ Mean percentage of BRCA2-protein levels in whole cell lysates. Full data are presented in Figure 2B.

^e^ Mean percentage of remaining RAD51 foci one hour following 4 Gy irradiation and hyperthermia, relative to the number of foci at 37 °C. Full data are presented in Figure 4C and Supplemental Figure 2A.

^f^ Mean percentage of integrated density of RAD51 foci one hour following 4 Gy irradiation and hyperthermia, relative to the number of foci at 37 °C. Full data are presented in Figure 4C and Supplemental Figure 2B.

1. Dewey WC, Hopwood LE, Sapareto SA, Gerweck LE. Cellular responses to combinations of hyperthermia and radiation. Radiology. 1977; 123: 463–74.

2. van Rhoon GC. Is CEM43 still a relevant thermal dose parameter for hyperthermia treatment monitoring? Int J Hyperthermia. 2016; 32: 50–62.
